# NG2 cells (polydendrocytes) in brain physiology and repair

**DOI:** 10.3389/fnins.2014.00133

**Published:** 2014-06-27

**Authors:** Akiko Nishiyama, Ryusuke Suzuki, Xiaoqin Zhu

**Affiliations:** Department of Physiology and Neurobiology, University of ConnecticutStorrs, CT, USA

**Keywords:** NG2, polydendrocyte, oligodendrocyte, myelin, demyelination, cell fate, subventricular zone

## Abstract

NG2 cells, also referred to as oligodendrocyte precursor cells (OPCs) or polydendrocytes, represent a major resident glial cell population that is distinct from mature astrocytes, oligodendrocytes, microglia, and neural stem cells and exist throughout the gray and white matter of the developing and mature central nervous system (CNS). While their most established fate is the oligodendrocyte, they retain lineage plasticity in an age- and region-specific manner. During development, they contribute to 36% of protoplasmic astrocytes in the ventral forebrain. Despite intense investigation on the neuronal fate of NG2 cells, there is no definitive evidence that they contribute substantially to the neuronal population. NG2 cells have attributes that suggest that they have functions other than to generate oligodendrocytes, but their exact role in the neural network remains unknown. Under pathological states, NG2 cells not only contribute to myelin repair, but they become activated in response to a wide variety of insults and could play a primary role in pathogenesis.

## Introduction

NG2 cells represent a resident glial progenitor cell population that exists throughout the gray and white matter of the developing and mature mammalian central nervous system (CNS) and are distinct from astrocytes, mature oligodendrocytes, microglia, and neural stem cells (reviewed in Nishiyama et al., 2009; Hill and Nishiyama, 2014). Their widespread existence in the CNS began to be recognized in the 1990s by immunohistochemical labeling for NG2 and the alpha receptor for platelet-derived growth factor (Pdgfra). Currently, NG2 cells are considered as the fourth major glial cell type in the CNS, comprising 2–8% of all the cells in the adult CNS (Dawson et al., 2003; Peters, 2004). These cells are often equated with oligodendrocyte precursor cells (OPCs) because of their ability to generate myelinating and non-myelinating oligodendrocytes. However, not all NG2 cells differentiate into oligodendrocytes, and oligodendrocytes are not their only fate, as discussed below. Different names have been used to refer to these cells. The term OPCs is used when discussing their role in oligodendrocyte production, while the terms “NG2 cells” and “NG2 glia” are used when discussing their property that is not directly related to their OPC role, even though NG2, which is also expressed on vascular pericytes, is not an absolute marker for these cells. To avoid using different names to refer to the same cells in different biological contexts, the word “polydendrocytes” has been suggested as a unified name for these cells, in keeping with the names of other types of glia that are loosely associated with their morphology. This perspective article will discuss recent findings and unsolved questions related to the astrocyte and neuronal fate of NG2 cells and their role in brain pathophysiology, primarily in the rodent CNS.

## The fate of NG2 cells

**Figure 1:**
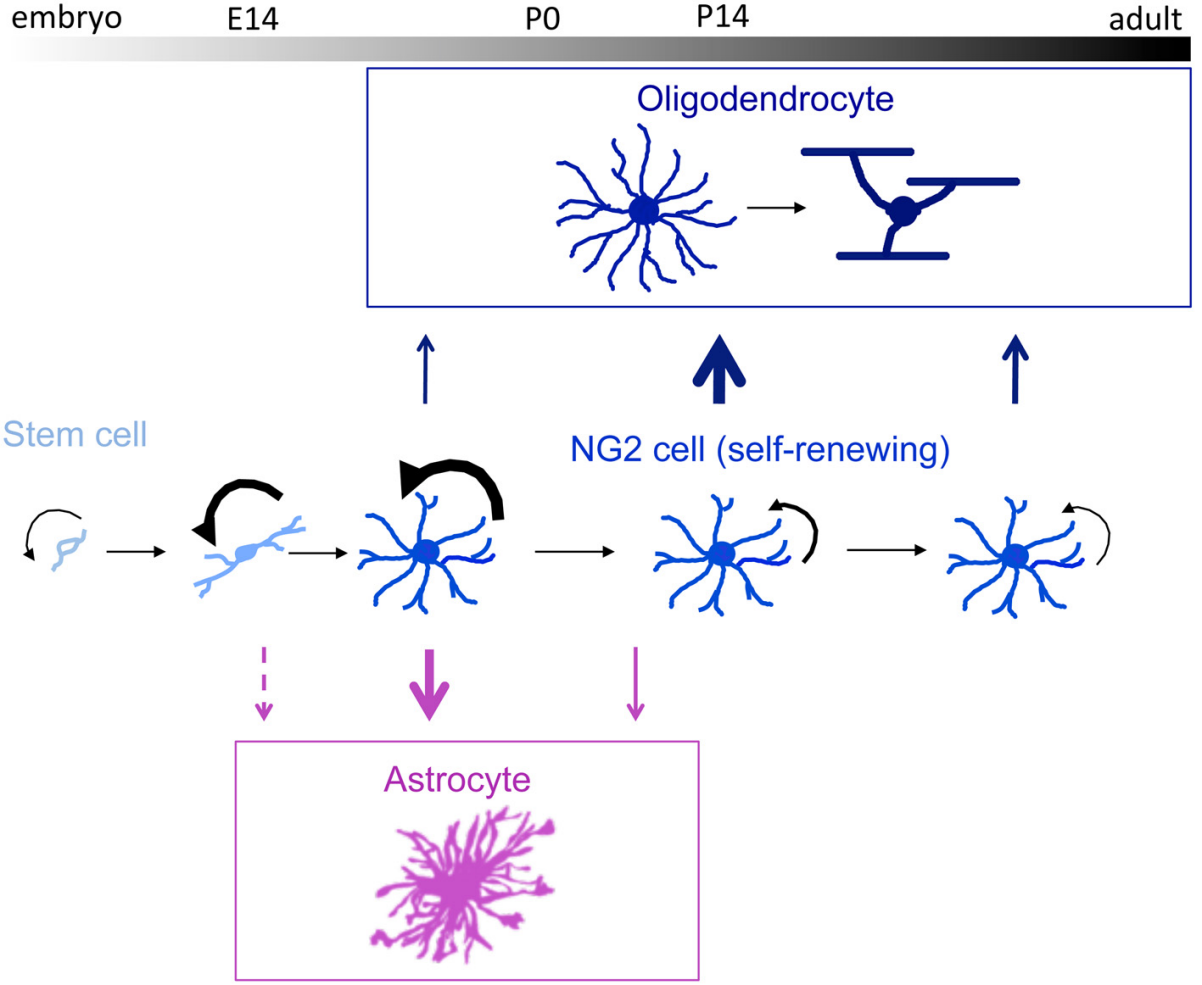
**A schematic showing the fate of NG2 cells throughout development**. The middle diagram depicts self-renewing NG2 cells, with a chronological scale across the top. Black arrows indicate the rate of proliferation, which is greatest perinatally. Blue upward arrows indicate oligodendrocyte differentiation, peaking during the third postnatal week. Purple downward arrows indicate astrocyte differentiation, which occurs predominantly before birth and gradually ceases shortly after birth. The thickness of the arrows denotes the extent of differentiation. Modified from Nishiyama (2007).

NG2 cells expand their population by extensive self-renewal. After their peak proliferation during the perinatal period, they retain their proliferative ability throughout life (Figure 1).

### Oligodendrocyte fate

The general consensus from a series of Cre-loxP-mediated genetic fate mapping studies is that under normal physiological conditions, NG2 cells in the adult CNS generate oligodendrocytes as they continue to self renew (Figure 1) (Dimou et al., 2008; Rivers et al., 2008; Kang et al., 2010; Zhu et al., 2011; Young et al., 2013). It is unlikely, however, that every NG2 cell will differentiate into an oligodendrocyte at some point during the life of the animal, as their uniform distribution does not parallel the distribution of oligodendrocytes (Dawson et al., 2000; Tomassy et al., 2014). It remains to be explored whether all NG2 cells are equivalent in their ability to generate oligodendrocytes or whether there is a subpopulation that is fated to permanently remain as NG2 cells.

### Astrocyte fate and lineage plasticity of NG2 cells

During development, the fate of NG2 cells is not restricted to oligodendrocytes. NG2 cells contribute to 40% of the protoplasmic astrocytes in the gray matter of the ventral forebrain (Zhu et al., 2008, 2011; Huang et al., 2014). The magnitude and the temporal and spatial distribution of protoplasmic astrocytes observed in these studies are quite distinct from the other observations where a small number (1~5%) of sporadically distributed reporter+ astrocytes were seen from Olig2-creER and Pdgfra-CreER fate mapping in adult (Dimou et al., 2008; Tripathi et al., 2010). Two independently generated tamoxifen-inducible NG2-creER transgenic mouse lines (BAC transgenic and knock-in) indicate that astrocyte generation from NG2 cells is most robust prenatally and tapers off during the first postnatal week (Figure 1) (Zhu et al., 2011; Huang et al., 2014), consistent with the chronology of astrocyte development. This suggests that the astrocyte fate of NG2 cells is a physiological developmental function and not due to radial glial expression of NG2 as suggested in Richardson et al. (2011). None of the other fate mapping studies had attempted to induce Cre prenatally. The reason why NG2 cells lose their astrogliogenic ability shortly after birth could be due to a density-dependent mechanism that regulates astrocyte production (Nakatsuji and Miller, 2001; Zhu et al., 2012).

Are NG2 cells that do not generate astrocytes during early development permanently committed to generating oligodendrocytes? The following observation suggests that under normal conditions, they are restricted to the oligodendrocyte lineage, but that they retain the ability to become astrocytes under certain conditions. When the oligodendrocyte transcription factor Olig2 that is required for NG2 cell specification (Rowitch, 2004; Richardson et al., 2006), was deleted in all NG2 cells, there was a complete fate switch from oligodendrocytes to astrocytes in the neocortex but not in the ventral forebrain, resulting in severe hypomyelination (Zhu et al., 2012). When Olig2 was deleted in early postnatal NG2 cells, only 50% of the Olig2-deleted neocortical NG2 cells switched their fate to astrocytes (Zhu et al., 2012). In the adult, deletion of Olig2 did not convert them into astrocytes, even in response to a stab wound (Komitova et al., 2011), nor was there increased astrocyte generation from NG2 cells in Olig2-creER heterozygous mice (Dimou et al., 2008), although the former study had used an inefficient Cre reporter. Thus, lineage restriction of NG2 cells appears to occur gradually during the first few postnatal weeks. Even after oligodendrocyte specification has occurred during embryogenesis, NG2 cells in certain regions retain some degree of context-dependent lineage plasticity, which is gradually lost in later postnatal life.

### Neuronal fate of NG2 cells

The neuronal fate of NG2 cells has been one of the most highly debated topics, and Cre-loxP-mediated genetic fate mapping studies have produced inconsistent findings. For example, two studies using Pdgfra-CreER or PLP (proteolipid protein)-CreER transgenic mouse lines observed reporter+ neurons in the piriform cortex (Rivers et al., 2008; Guo et al., 2010), while a subsequent study using an independent line of Pdgfra-CreER mice did not find any evidence for a neuronal fate (Kang et al., 2010). Earlier studies using NG2- and Olig2-Cre driver mice showed no evidence for neurogenesis (Dimou et al., 2008; Zhu et al., 2008; Komitova et al., 2009; Zhu et al., 2011), while a recent study using the same NG2-creER mice showed a few reporter+ neurons in the hypothalamus (Robins et al., 2013). What is the significance of detecting reporter+ neurons in these fate mapping studies? Is the extent of neurogenesis from NG2 cells sufficiently large to bring about a physiological effect in the neural network? The findings must be interpreted in proper context without overemphasizing observations where sporadic reporter+ neurons are found.

**Figure 2:**
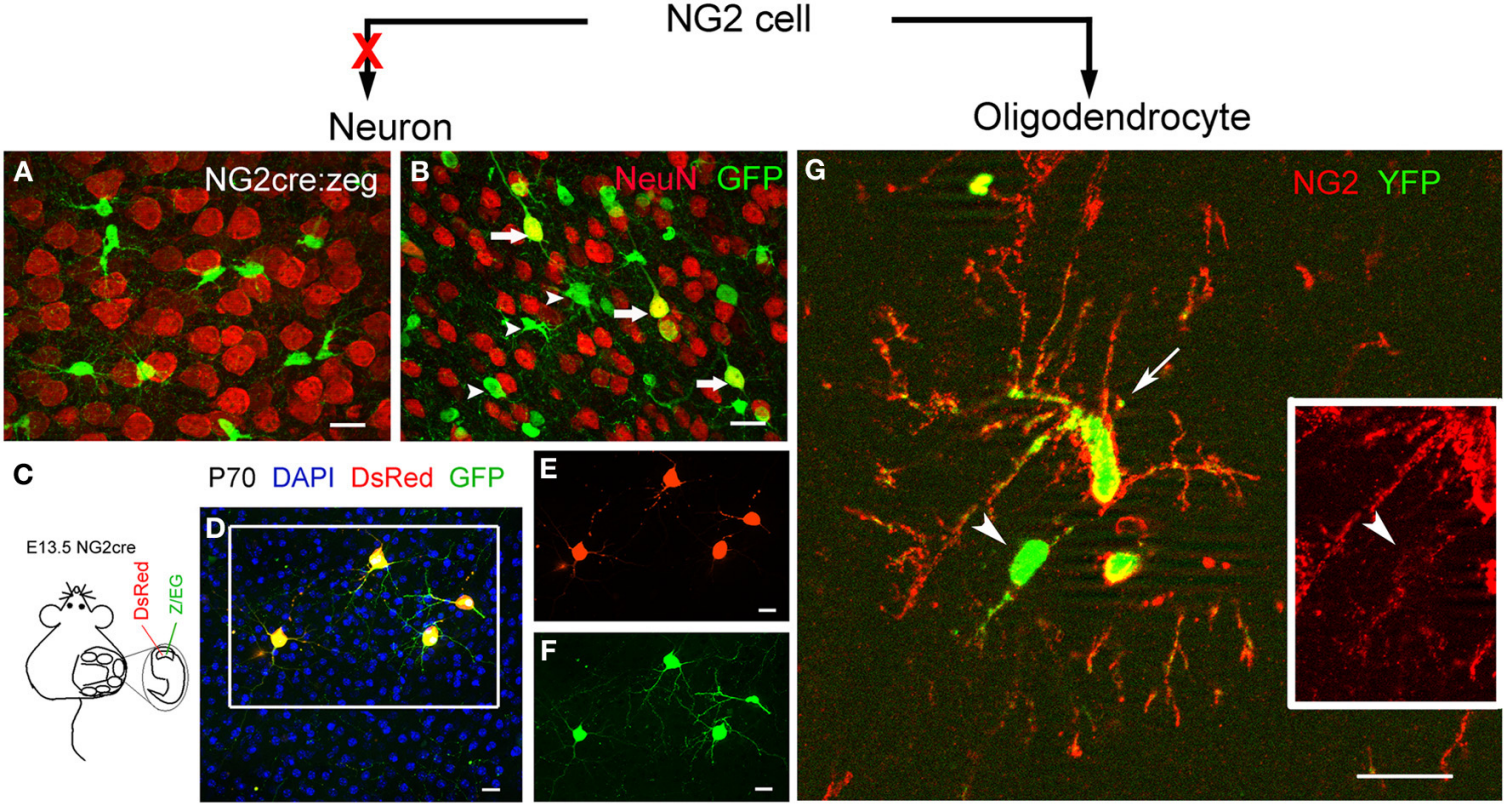
**Lineage-dependent and independent reporter expression in Cre-loxP fate mapping. (A,B)** Double labeling for GFP and the neuronal marker NeuN in the neocortex of NG2cre:zeg mice at P14 **(A)** and P70 **(B)**. NeuN+ GFP+ cells with mature neuronal morphology (arrows) are seen as well as NeuN- GFP+ NG2 cells (arrowheads) in P70 but not in P14 cortex. **(C–F)** Transfection of zeg reporter plasmid into neuronal precursor cells in NG2cre single transgenic mice. **(C)** Scheme showing co-transfection of DsRed and zeg plasmid DNA into the lateral ventricles of E13.5 NG2cre single transgenic mice by in utero electroporation and positioning the electrodes to target the dorsal pallium. **(D–F)** GFP expression in electroporated neurons at P70. All the GFP+ cells were neurons that had been electroporated (DsRed+), and no GFP+ glial cells were detected. This experiment demonstrates that Cre is activated directly in neocortical neurons of NG2cre mice at some point between P14 and P70, leading to GFP expression in neurons. Unlike the case for oligodendrocytes shown in G, there were no GFP+ cells that appeared to be in transition from NG2 cells to neurons. **(G)** Corpus callosum from P70 NG2creER:YFP mice 14 days after Cre induction with 4-hydroxytamoxifen. A cell that appears to be transitioning from an NG2 cell (NG2+ YFP+) into an NG2- oligodendrocyte (NG2- YFP+) with weak NG2 immunoreactivity in the processes (arrowheads) is seen next to a typical NG2 cell that robustly expresses NG2 (arrow). Inset shows single labeling of the cell marked by arrowhead for NG2.

The following example illustrates one of the caveats of the Cre-loxP technology that spurious transient activation of Cre in an unrelated cell could lead to reporter expression in that cell in the absence of lineage progression. In NG2-cre:zeg mice generated by crossing constitutively active NG2-cre mice (Zhu et al., 2008) to the zeg reporter mice (Novak et al., 2000), a significant number of reporter+ neurons appeared in the neocortex after P45 but not at P14 (Figures 2A,B). To determine whether reporter+ neurons arose as a result of lineage progression from NG2 cells or due to direct Cre expression in neurons, the zeg reporter plasmid was in utero electroporated directly into neuronal precursors of NG2-cre single transgenic mice at E13.5, and the appearance of reporter+ neurons was examined (Figure 2C). The plasmid would be retained in neurons that are undergoing their last cell division and lost from glial cells as they undergo multiple divisions (Bai et al., 2003). The expression of the reporter in neurons would suggest direct Cre activation in neurons. A DsRed plasmid was co-electroporated to mark the transfected cells. When the electroporated mice were sacrificed at P70, all the DsRed+ neurons also expressed EGFP (Figures 2D–F), and no NG2 cells expressed EGFP or DsRed. This suggests that there was transient Cre (and possibly NG2) expression in neurons, and that the duration of Cre expression was sufficient for Cre-mediated recombination to allow EGFP expression from the zeg plasmid but not sufficiently long-lasting to be detected by Cre immunohistochemistry or in situ hybridization. It is possible that certain physiological conditions cause a spike in NG2 transcription, which is too transient to be detected in NG2-DsRed transgenic mice (Zhu et al., 2008). Furthermore, no transitional forms between NG2 cells and neurons could be observed, unlike the case for NG2 cells transitioning into astrocytes (Zhu et al., 2012) or oligodendrocytes (Figure 2G).

In the new NG2 cell fate mapping study using the NG2creER knockin mice crossed to ROSA-tdTomato reporter, Huang et al. (2014) observed reporter+ neurons with electrophysiological and morphological properties of neurons in the non-neuronogenic regions of the forebrain. By contrast, very few reporter+ neurons were found when the same mice were crossed to the less efficient RSOA-YFP reporter. Since these neurons appeared without evidence of proliferation, it is unlikely that they arose from NG2 cells, as previously reported (Clarke et al., 2012), but rather by some form of Cre-dependent DNA recombination that had occurred in neuronal cells. The Huang study also highlights the different outcomes of studies using Cre reporter lines with different efficiencies. Development of a novel, Cre-independent method is needed to resolve the question of the neuronal fate of NG2 cells.

## Relationship of NG2 cells to neural stem cells

The adult SVZ consists of a heterogeneous population including GFAP+ neural stem cells (type B cells), transit-amplifying cells (type C cells), and neuroblasts that migrate to the olfactory bulb via the rostral migratory stream (type A cells) (Gonzalez-Perez and Alvarez-Buylla, 2011). Many early studies were focused on testing the then attractive hypothesis that NG2 cells corresponded to multipotential neural stem cells in the SVZ (Nunes et al., 2003; Aguirre and Gallo, 2004), based on the observation that they could be induced to differentiate into astrocytes and neurons under certain culture conditions (Roy et al., 1999; Kondo and Raff, 2000). However, further examination of NG2 cells and the SVZ revealed that NG2 cells comprise a minority of cells, located mostly at the periphery of the SVZ, and are distinct from the Dlx2-expressing type C cells or neuroblasts that express Doublecortin (Komitova et al., 2009; Platel et al., 2009; Richardson et al., 2011). These studies also showed that NG2 cells are distinct from GFAP+ neural stem cells (type B cells) (Rivers et al., 2008; Komitova et al., 2009; Chojnacki et al., 2011), in contrast to an earlier study that showed expression of Pdgfra on type B cells (Jackson et al., 2006). Neural stem cells do generate NG2 cells, but this fate of neural stem cells seems to be a minor fate compared with their neurogenic fate and is highly region-specific. Interestingly, a recent real-time imaging study of the fate of single cells unequivocally demonstrated that neural stem cell clones that generate NG2 cells do not generate neurons and are primarily found in the dorsal SVZ, while those that generate neurons are more enriched in the lateral SVZ and do not generate NG2 cells (Ortega et al., 2013). Thus, there appears to be an early segregation of neuronal and oligodendrocyte lineages in the SVZ. Under normal conditions, only SVZ type C cells, but not NG2 cells, proliferate in response to epidermal growth factor (EGF) (Doetsch et al., 2002; Hill et al., 2013). However, under pathological conditions such as EGF overexpression or demyelination, EGF can redirect SVZ type C cells to become NG2 cells (Aguirre et al., 2007; Ivkovic et al., 2008; Jablonska et al., 2010; Galvez-Contreras et al., 2013). These observations can be explained if EGF receptor becomes upregulated on a small population of cells that are in transit from SVZ type C cells to becoming NG2 cells.

## The role of NG2 cells in the normal CNS

Why has the mammalian brain evolved to maintain such a uniformly distributed glial cell type? Recent studies have revealed that new oligodendrocytes and myelin continue to be produced in the mature CNS (Zhu et al., 2011; Young et al., 2013) and a significant amount of activity-dependent myelin plasticity occurs in the adult (Zatorre et al., 2012; Hill and Nishiyama, 2014). NG2 cells also generate non-myelinating perineuronal oligodendrocytes whose somata lie apposed to neuronal somata (Penfield, 1924). Although the role of the perineuronal oligodendrocytes is not clear, they can produce myelin in response to demyelination (Ludwin, 1979) and could be providing neurotrophic and metabolic support for neurons (Taniike et al., 2002; Fünfschilling et al., 2012; Lee et al., 2012).

NG2 cells are evenly distributed to cover the entire mature CNS parenchyma (Dawson et al., 2000). *In vivo* imaging in 2–3-month-old neocortex revealed non-overlapping territories occupied by adjacent NG2 cells, and their processes appeared to be contact-inhibited (Hughes et al., 2013). Another study using fixed hippocampi from 3–4-week-old rats showed that NG2 cells were tiled but shared approximately 5% of the volume with adjacent NG2 cells (Xu et al., 2013). It is not clear whether the extent of overlap between processes of neighboring NG2 cells changes as the brain matures. Regardless, the uniform distribution of NG2 cells would suggest a yet uncovered homeostatic role in the CNS.

NG2 cells interact uniquely with neurons in that they depolarize in response to receiving direct synaptic input from neuronal axons (Bergles et al., 2000). However, the extent of depolarizations is not sufficient to elicit repetitive action potentials, and thus NG2 cells are still considered as non-excitable glial cells. While the physiological consequences and significance of neuron-NG2 cell synapses remain unknown, and the nature of neuron-NG2 cell communication changes with age and differentiation (Maldonado and Angulo, 2014), it is likely that local increases in intracellular calcium play an important role in mediating downstream cellular effects (Bergles et al., 2000; Ge et al., 2006; Hamilton et al., 2010; Haberlandt et al., 2011).

## The role of NG2 cells in pathology

### Repair of demyelinating lesions

It is well established that NG2 cells proliferate and differentiate into myelinating oligodendrocytes and repair demyelinated lesions (Di Bello et al., 1999; Watanabe et al., 2002; Tripathi et al., 2010). It still remains to be shown whether replenishment of the NG2 cell population can be a cause for remyelination failure under certain conditions. While repeated acutely demyelinated lesions undergo successful remyelination (Penderis et al., 2003), other studies suggest that NG2 cells can become depleted after acute demyelination (Keirstead et al., 1998) and their repopulation may not occur fast enough to meet the demands of chronic ongoing demyelination (Mason et al., 2004). Recruitment of new NG2 cells could occur by proliferation of local NG2 cells and/or migration and differentiation of cells from the SVZ (Nait-Oumesmar et al., 1999; Picard-Riera et al., 2002; Etxeberria et al., 2010; Tepavcevic et al., 2011). However, evidence is not yet strong that these SVZ-derived cells are capable of fully differentiating into remyelinating cells to the extent that local NG2 cells are.

### Activation of NG2 cells in other types of lesions

NG2 cells undergo increased proliferation and dramatic morphological changes in response to a wide variety of acute CNS insults besides demyelination, including spinal cord injury (McTigue et al., 2001; Jones et al., 2002), ischemia (Zhang et al., 2013), excitotoxic injury (Bu et al., 2001; Wennström et al., 2004), and viral infection (Levine et al., 1998). The time course of their “activation” and their “reactive morphology” or the extent of proliferation varies depending on the nature of the insult, but the functional significance for these diverse morphological and proliferative changes is not known. For example, it is not known whether the shorter, thicker processes reflect increased uptake of extracellular fluid/ions or increased phagocytic activity. Nor is it known whether the increased number of thin, elongated process after viral infection reflect a search for something or deregulated cytoskeleton. *In vivo* imaging has revealed that NG2 cell processes are highly dynamic (Hughes et al., 2013; Hill et al., under revision), but it is not known what they are seeking besides axons to myelinate.

In most cases of acute injury, NG2 cell responses occur early, within 24 h (Watanabe et al., 2002; Horky et al., 2006; Simon et al., 2011), which is similar to or slightly lags behind the time course of microglial response and a few days before reactive astrogliosis becomes apparent. Some forms of insult such as excitotoxic injury seem to elicit a greater microglial response than NG2 cell response. NG2 cells exhibit a close spatial relation to astrocyte processes and microglial somata (Nishiyama et al., 2002; Hamilton et al., 2010; Xu et al., 2013), and the latter becomes more pronounced in response to injury (Nishiyama et al., 1997; Bu et al., 2001; Wu et al., 2010). Future studies can be directed to studying how the three types of reactive glia signal to each other to achieve a concerted response specifically tailored to each type of injury.

### NG2 cells in pathogenesis

The inherent ability of NG2 cells to remain in cell cycle through life also makes them susceptible to neoplastic transformation. Although the cell of origin of glioblastoma multiforme continues to be debated, cell fate mapping of neural stem cells or NG2 cells with deletions in p53 and NF1 genes revealed that neoplastic changes begin to occur in NG2 cells and not in neural stem cells (Liu et al., 2011). Intriguingly, early proliferative foci arise in perineuronal locations in gray matter rather than in white matter tracts where glioma cells are known to expand and disseminate, suggesting a proliferative paracrine signal imparted by neurons.

Several recent studies have shown that metabolic defects in oligodendrocytes can precede neurodegeneration in amyotrophic lateral sclerosis (ALS) (Lee et al., 2012; Kang et al., 2013; Philips et al., 2013), strongly suggesting a pathogenic role for oligodendrocytes. In addition, a direct pathogenic role for NG2 cells has been shown at the neurovascular interface. In a cerebral hypoperfusion model, metalloproteinase-9 (MMP-9) is secreted from NG2 cells in the vicinity of vascular endothelial cells, leading to degradation of the endothelial tight junction protein ZO-1 and breakdown of the blood-brain barrier (Seo et al., 2013). These findings highlight the importance of the oligovascular niche in normal and pathological conditions that could be an important topic of future investigations (Maki et al., 2013; Miyamoto et al., 2014).

### Conflict of interest statement

The authors declare that the research was conducted in the absence of any commercial or financial relationships that could be construed as a potential conflict of interest.
